# pH-responsive superstructures prepared via the assembly of Fe_3_O_4_ amphipathic Janus nanoparticles

**DOI:** 10.1093/rb/rby016

**Published:** 2018-07-12

**Authors:** Shuang Cai, Bin Luo, Xiaohui Zhan, Xiaoxi Zhou, Fang Lan, Qiangying Yi, Yao Wu

**Affiliations:** National Engineering Research Center for Biomaterials, Sichuan University, Chengdu, Sichuan, P.R. China

**Keywords:** Janus Fe_3_O_4_ nanoparticles, Co-assembly, pH-sensitive, Controlled release

## Abstract

The strategy of using Fe_3_O_4_ amphiphilic Janus nanoparticles (Fe_3_O_4_@AJNPs) bearing β-cyclodextrin (β-CD) and aminopyridine (APD) functionalized polymethyl methacrylate (PGMA) to construct pH-stimuli responsive co-assemblies through host-guest interactions between β-CD and APD was proposed. The spherical co-assemblies with an average diameter about 210 nm were excellent magnetic responsive and quite stable even up to 2 months in deionized water. The pH-liable capability of these co-assemblies was revealed by disassembly of the formed superstructures with destruction of the built inclusion complexes. The disassembly process was monitored by SEM, TEM, DLS and fluorescent molecules probe. After disassembly of the co-assemblies caused by protonation of nitrogens in APD, hydrophobic PGMA-APD lacking of interactions with the Fe_3_O_4_@AJNPs chains was precipitated, and the remained Fe_3_O_4_@AJNPs turned to re-assemble to self-assemblies. Besides, the recyclable Fe_3_O_4_@APJNs could reassembly with additional PGMA-APD to build co-assemblies with a uniform morphology for several times. These pH-sensitive co-assemblies with high stability, good magnetic responsiveness and cytocompatibility could be used as pH-responsive vehicles within which to encapsulate drugs for subsequent controlled release.

## Introduction

Janus particles are a special type of colloidal particles with different building architectures on their two opposite hemispheres. They have attracted tremendous research interests in the field of chemical synthesis, catalysis, biomedical applications, etc. [1–4]. Due to their isotropic surface chemical compositions, Janus particles can be endowed with features unique to their individual hemispheres. Typically, the amphiphilic structures of Janus particles made them well known as surfactants due to their hydrophilic components on one hemisphere and hydrophobic components on the other [5–7]. Taking advantages of such amphiphilic property, stabilization/immobilization of two immiscible phases was possible, and thus engineering of superstructures with controllable location of functional components could be easy achieved. For example, Passas-Lagos et al., reported the iron-based Janus particles with mushroom-like morphologies, of which Fe_3_O_4_ nanoparticles were immobilized and further magnetic manipulation for desired application [8]. Wang et al., controlled the location of active species (fluorescent dye and catalytic molecules) in the bi-compartments composed of Janus particles for release of small substances across aqueous-organic phase [9].

Just like the wide investigated assembly of homogeneous colloidal particles, Janus particle with specially designed architectures and distinct physicochemical properties are capable to assemble into superstructures [10–12]. As a consequence, advanced properties of the individual building blocks could be preserved, and most importantly, new features that cannot be monitored in the assemblies of homogeneous particles could be created. For example, self-assembly of the particulate Au NPs decorated with bicompartmental brushes of polymethyl methacrylate (PMMA) and polyethylene oxide (PEO) in dioxane could obtain long chain-like structures [13]. Moreover, tertiary wormlike assemblies with regularly spaced disks containing well arrays of cadmium sulfide (CdS) nanoparticles could be prepared through the assembly of polystyrene-block-poly (acrylic acid)-block poly (methyl methacrylate) (PS-b-PAA-b-PMAA) triblock copolymer brushes modified CdS nanoparticles in water [14].

The study of Janus particle assembly provides more general fundamental understanding on self-assembly [15]. Actually, in recent years, the manufacture of ordered superstructures of inorganic nanoparticles through self-assembly is the most promising trend. With respect to the advantages mentioned above, the most commonly used assembly strategies were based on mutual attraction of the hydrophobic parts of the Janus particles [12]. However, self-assembly of these amphiphilic Janus nanoparticles into ordered superstructures still remain challenging [2, 16]. Owing to the relatively weak particle–particle interactions generated by the mutual attraction of the hydrophilic or hydrophobic parts, the amphiphilic Janus nanoparticles are inclined to escape from the attraction and rearrange of the superstructures [17, 18]. Superstructures obtained by the assembly of amphiphilic Janus nanoparticles possess no definite shapes, instead, they wriggle and change their con with time [19]. Moreover, it is very difficult to endow the assemblies with multi-functions and stimuli-responsive capability, which are limited the further biomedical applications [20, 21]. To activate the stable Janus assemblies for desired applications, in addition to external triggering signal, for example the magnetic field, for remote manipulation of the assembled superstructures, the stronger and switchable interactions between Janus particles would be preferred. In our previous study, we reported the morphology switchable magnetic co-assemblies [22]. Entanglement interactions between free polymers and chemically modified Fe_3_O_4_ NPs facilitated fabrication of the co-assemblies and the pH sensitive poly (4-viny pyridine) segment further enabled adjustment of the assembled system via environmental pH variation. Inspired by this, we proposed the strategy of using Janus particles together with functional polymers to construct stimuli-responsive co-assemblies. In particular, the famous host-guest interactions based on superamolecular binding between β-cyclodextrin (β-CD) and pyridine ring could be employed as the pH-sensitive binding. It was suggested that β-cyclodextrin (β-CD) pyridine ring could form 1:1 inclusion complex with pyridine ring in neutral or alkaline environment, due to the stronger hydrogen bond and proper size of the host and guest molecules [23]. Accordingly, lower the environmental pH value to less than 7 could induce protonation of the pyridine nitrogen, leading to disassembly of the complexes [24, 25].

Herein, the application of host-guest interactions as pH-responsive binding for fabrication of stimuli-responsive Janus particle assemblies was demonstrated. In general, to prepare the superparamagnetic Fe_3_O_4_ amphiphilic Janus nanoparticles (Fe_3_O_4_@AJNPs), the PEG was used to moderate the Fe_3_O_4_ nanoparticles through ‘Graft to’ method to get the hydrophilic hemisphere, while PMMA-MA moderated with β-CD was used to get the hydrophobic hemisphere. Firstly, the Fe_3_O_4_@AJNPs had excellent amphipathic structures which made them self-assembly to form shuttle-like self-assemblies in aqueous phase. The additional polymer PGMA with branched pyridine ring could work together with Fe_3_O_4_@AJNPs to construct co-assemblies with more orderly and stable spherical superstructures and pH-responsive behavior through the host-gust interactions between β-CD and APD. These pH-sensitive co-assemblies with good magnetic responsiveness and cytocompatibility would make it possible to provide promising candidates loaded-drug for controllable release.

## Materials and experiments

**Scheme 1:**
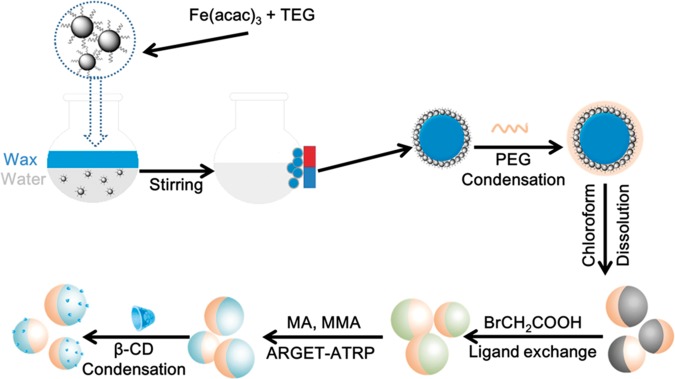
Schematic representation for the synthesis of Fe_3_O_4_@AJNPs

In this report, we combined previous study and a ‘Graft to’ method [26] to build up Fe_3_O_4_@AJNPs (Scheme 1).

### Materials

Foetal bovine serum (FBS), penicillin, streptomycin and Dulbecco’s minimum essential medium (DMEM) and were purchased from HyClone (USA). A Cell counter kit-8 (CCK-8) was purchased from Dojindo Laboratories (Kumamoto, Japan). The NIH 3T3 cell line and HepG2 cell line (Human hepatoma carcinoma cell) were purchased from the Chinese Academy of Science Cell Bank for Type Culture Collection (Shanghai, China). Fe(acac)_3_, triethylene glycol (TEG), APD, thionyl chloride, 4-dimethylaminopyridine (DMAP), N, N′-dicyclohexylcarbodiimide (DCC), N, N-dimethylformamide (DMF) and bromoacetic acid, hydroxypropyl-β-cyclodextrin (β-CD) were purchased from Sigma Aldrich. Methyl methacrylate (MMA) and methacrylic acid (MA) were purchased from TCI. Paraffin wax, dichloromethane, HOOC-PEG2000, PGMA_35_, dimethylsulfoxide (DMSO) and doxorubicin (DOX) were purchased from other commercial ways.

### Synthesis of amphipathic Janus Fe_3_O_4_ nanoparticles (Fe_3_O_4_@AJNPs)

#### Synthesis of the hydrophilic Fe_3_O_4_ nanoparticles (Fe_3_O_4_ NPs)

Hydrophilic superparamagnetic Fe_3_O_4_ NPs were prepared via the polyol method [27]. Briefly, Fe(acac)_3_ (720 mg) was mixed with TEG (40 ml) under magnetic stirring. The mixture was first heated to 180°C for 30 min and then was heated to 280°C for another 30 min under nitrogen protection. The suspension was obtained after cooling to room temperature.

#### Synthesis of Fe_3_O_4_/wax composite microsphere

The theory of Pickering emulsions was employed to obtained emulsions of Fe_3_O_4_/wax microspheres. Briefly, paraffin wax (540 mg) and 20 ml of ultrapure water were added to above Fe_3_O_4_ suspension (10 ml) with mechanical stirring at 95°C for 1.5 h. When the products were cooled to room temperature, they were washed with ethyl alcohol for several times in order to remove the weakly attached magnetic particles and non-magnetic particles. Then ethyl alcohol was replaced by using DMF to wash Fe_3_O_4_/wax composite microspheres for several times. Finally, the desired Fe_3_O_4_/wax composite microspheres were dispersed in 5 ml anhydrous DMF.

#### Synthesis of surface-initiated Fe_3_O_4_ NPs (Fe_3_O_4_−PEG NPs)

PEG (2000)-COOH was reacted with Fe_3_O_4_ NPs through the condensation reaction between carboxyls and hydroxyls using a ‘Graf-to’ method. PEG-COOH (500 mg) was dissolved in 8 ml anhydrous methylene chloride. Thionyl chloride (2 ml) was trickled into above solution with mechanical stirring at 25°C for 6 h. After removing the solvent and the rest of the thionyl chloride by reduced pressure distillation, the oil-like acyl chloride PEG2000 was obtained and dissolved in 2 ml anhydrous DMF. Then acyl chloride PEG (2 ml), DMAP and DCC with a molar ratio of 1:2:2 were added into Fe_3_O_4_/wax composite microspheres anhydrous DMF solution (5 ml). The mixtures were reacted under the nitrogen protection at room temperature for 12 h. The final PEG–Fe_3_O_4_/wax composite microspheres were separated using magnets and washed with deionized water for several times. The PEG–Fe_3_O_4_/wax composite microspheres were then washed with chloroform to dissolve the wax. The final Fe_3_O_4_–PEG NPs were separated by a commercial magnet.

#### Synthesis of PEG–Fe_3_O_4_–Br

The PEG–Fe_3_O_4_ dispersed in 5 ml ethanol was then injected into the light-resistant container with bromoacetic acid (300 mg) under mechanical stirring at room temperature for 48 h. The final PEG–Fe_3_O_4_–Br NPs product separated using magnets was washed with ethyl alcohol to remove the unreacted bromoacetic and dispersed in DMF (2 ml).

#### Synthesis of PEG–Fe_3_O_4_–PMMA–MA (CD) (Fe_3_O_4_@AJNPs)

Sodium ascorbate (5.2 mg, 26 μmol) in a closed light-resistant container was firstly vacuumized and infiltrated with nitrogen. Then MMA (201 μl), MA (201 μl) and Cu^2+^/PMDETA solution (mixture of 44.3 mg CuBr_2_, 417.9 μl PMDETA and 10 ml DMF) (390 μl) were sequentially injected into the closed container. Finally, above mentioned PEG–Fe_3_O_4_–Br NPs (2 ml) was injected into the reaction mixture with magnetical stirring at 70°C for 12 h. The final PEG–Fe_3_O_4_–PMMA–MA NPs were washed with DMF and dispersed in anhydrous DMF. Then β-CD was reacted with PEG–Fe_3_O_4_–PMMA–MA NPs through the condensation reaction between carboxyl (in PMMA-MA) and hydroxyls (in β-CD) under the protection of nitrogen at room temperature for 24 h. The finally Fe_3_O_4_@AJNPs were collected under an external magnetic field.

### Synthesis of PGMA-PD

PGMA_35_ (391 mg) dispersed in 5 ml anhydrous DMF was mixed with excess APD (200 mg) in light-resistant container under a dry nitrogen atmosphere at room temperature for 48 h. The excess APD was removed by dialysis against DMSO in a dialysis bag with cut-off Mw of 1000. Then, DMSO in the dialysis bag was replaced by water. The final product was obtained by freeze-drying.

### Self-assembly of Fe_3_O_4_@AJNPs and co-assembly of Fe_3_O_4_@AJNPs with PGMA-APD

Cosolvent method was carried out for self-assembly and co-assembly. Briefly, deionized water was added dropwise into the solution of 1 ml Fe_3_O_4_@AJNPs (3.5 mg) in DMF and 1 ml DMF mixture of Fe_3_O_4_ @AJNPs (3.5 mg) and PGMA-APD (2.76 mg) under vigorous agitation. After the volume of the solution reached 10 ml, the mixtures were shaking for 12 h. Then, DMF was removed by using dialysis in water using 3.5 kD molecular weight cut-off (MWCO) dialysis bags for 24 h. Finally, the self-assemblies and co-assemblies were acquired by a magnet and redispersed in pure water.

### DOX loading and pH responsive fluorescent detection

DOX was encapsulated in co-assemblies as the fluorescent molecule during the assembly process to monitor the disassembly process of the Fe_3_O_4_@AJNPs-based nanospheres in different pH environments. A DMF solution of DOX (0.35 mg) was added to a mixture containing Fe_3_O_4_@AJNPs and PGMA-APD during the co-assembly process. Then, the as-prepared DOX-loaded co-assemblies were magnetic separated to remove free DOX. The concentration of DOX-loaded co-assemblies was regulated to 1.5 mg ml^−1^ and then added into buffers with pH 7.4, 5.7 and 4.5, respectively. At determined time, the above solutions were taken for fluorescence measurement.

### Re-assembly experiment

The final re-assemblies recycled by magnetic separation were dissolved in 1 ml DMF and then mixed with additional PGMA-APD (2.76 mg) to construct new co-assemblies via above mentioned method.

### Characterization of materials

The zeta potential and size distribution of the samples were recorded via dynamic light scattering (DLS, Zetasizer Nano ZS90, Malvern Company). The morphologies of the samples were observed by scanning electron microscopy (SEM, Hitachi S-4800, Japan) and transmission electron microscopy (TEM, JEM-2010, Japan electronic). Fourier transform infrared spectra (FTIR, PE spectrometer) were recorded with the wave number in the range of 500–4000 cm^−1^. TGA curve was obtained using a simultaneous thermal analysis (STA 449 C Jupiter, NETZSCH). The heating process was conducted from room temperature to 700°C under a nitrogen atmosphere at a rate of 10°C min^−1^. ^1 ^H NMR spectra of PGMA-APD in DMSO were acquired by a BrukerAvanceII400 MHz NMR Spectrometer. The magnetization of the dried sample was measured by a vibrating sample magnetometer (VSM, Model BHV-525, Riken Japanese Electronics Company). Fluorescence intensity was measured by a fluorescence spectrometer (RF-5301PC Shimadzu, Japan).

### *In vitro* cytotoxicity

The 3T3 and HepG2 cell lines were cultured in DMEM medium containing 10% FBS and 1% antibiotics in an incubator with 5% CO_2_ at 37°C. The CCK-8 assay was employed to evaluate the *in vitro* cytotoxicity of the co-assemblies, self-assemblies and PGMA-PD. The cells were seeded in a 96-well plate and cultured in an incubator for 24 h with 5% CO_2_ at 37°C. The serial dilutions of above materials in DMEM were used to treat the 3T3 and HepG2 cell lines separately. After incubation for 24 h, the culture medium was replaced by 100 μl of medium containing the CCK-8 (volume fraction 10%) reagent. The cells were incubated at 37°C for 2 h. The absorbance at 450 nm was measured by a microplate reader. Cells cultured without materials treatment were used as the control group. The cell viabilities were calculated as follows:

Cell Viability (100%) = (ODtreated − OD_CCK8_)/(ODcontrol  − ODCCK8) × 100%

## Results and discussion

### Synthesis and characterization of Fe_3_O_4_@AJNPs and PGMA-APD

**Figure 1:**
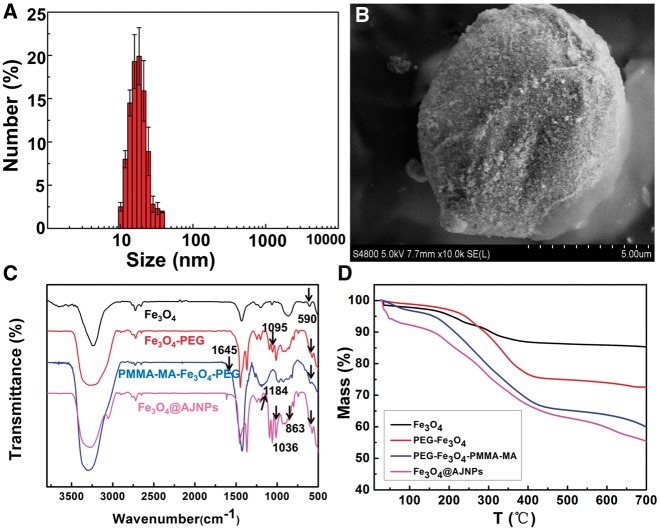
(A) Size distribution of hydrophilic Fe_3_O_4_ nanoparticles. (B) SEM image of the Fe_3_O_4_/wax composite microsphere. FTIR spectra (C) and TGA curves (D) of Fe_3_O_4_ NPs, PEG–Fe_3_O_4_ NPs, PEG–Fe_3_O_4_–PMMA–MA NPs and Fe_3_O_4_@AJNPs

The mean size of hydrophilic Fe_3_O_4_ NPs prepared by polyol process was 15.2 ± 2.1 nm as shown in Fig. 1A. The theory of Pickering emulsions was employed to obtain emulsions of Fe_3_O_4_/wax microspheres. It was clearly observed from SEM image that a homogeneous Fe_3_O_4_/wax composite sphere with a diameter of 6.5 ± 0.6 μm was independently scattered and Fe_3_O_4_ NPs with a mean diameter about 15.4 ± 1.9 nm were evenly distributed on the surface of wax (Fig. 1B). Then PEG (2000) terminated with carboxyl was used to react with the exposed hydroxyl groups on Fe_3_O_4_ NPs though ‘Graft to’ method to get Fe_3_O_4_–PEG NPs. Zeta potential of Fe_3_O_4_–PEG NPs was turned to be 2.3 mV, which was different from 7.3 mV of the initial Fe_3_O_4_ NPs due to the modification of electroneutral PEG chain. Meanwhile, as shown in Fig. 1C, the adsorption band at 590 cm^−1^ was assigned to the vibration of the Fe–O band. The new peak at 1095 cm^−1^ in infrared absorption spectrum of Fe_3_O_4_–PEG NPs was attributed to the absorption peak for PEG (C–O–C). All the above results suggested that PEG brush was successfully conjugated to the surface of Fe_3_O_4_ NPs [28]. The exposed hydroxyl groups on the opposite interface of PEG–Fe_3_O_4_ NPs were replaced with bromoacetic acid to obtain the PEG–Fe_3_O_4_–Br NPs via the ligand exchange reaction. The ARGET–ATRP was then applied to graft PMMA–MA brushes onto opposite hemisphere of PEG–Fe_3_O_4_–Br NPs to acquire the amphiphilic Janus PEG–Fe_3_O_4_–PMMA–MA. Infrared absorption spectrum of PEG–Fe_3_O_4_–PMMA–MA NPs was shown in Fig. 1C. The emerging absorption peak at 1645 cm^−1^ was attributed to the characteristic carbonyl absorption peak for PMMA–MA, suggesting the successful conjugation of PMMA–MA brushes to the surface of Fe_3_O_4_ NPs [29]. Hydroxypropyl-β-Cyclodextrin (β-CD) was reacted with PEG–Fe_3_O_4_–PMMA–MA through the condensation reaction between carboxyls (in PMMA-MA) and hydroxys (in CD) to obtain the final functionalized amphiphilic Janus PEG–Fe_3_O_4_–PMMA–MA (CD), which was also named as Fe_3_O_4_@AJNPs. As shown in Fig. 1C, the absorption peaks at 1184, 1036 and 863 cm^−1^ were attributed to the characteristic absorption peaks for β-CD, suggesting the successful conjugation of PMMA-MA with β-CD [30]. The mass ratio of the PEG, PMMA-MA brushes and β-CD to the final product were about 16.1%, 22.7% and 7.4%, respectively, calculated by data from TGA (Fig. 1D).

**Figure 2:**
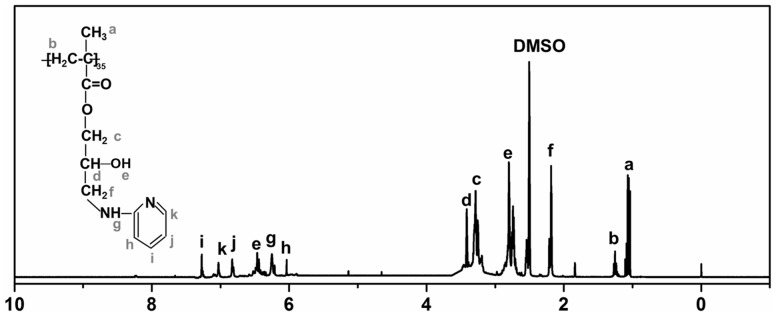
^**1**^H NMR spectrum of PGMA–APD (solvent: DMSO)

PGMA-PD was synthesized by PGMA with excess 2-aminopyridine. ^1^HNMR spectrum of PGMA-PD was shown in Fig. 2. The chemical shifts at 1.04, 1.09 and 1.19 ppm (a) were assigned to the protons of the methyl and the peaks at 1.29 ppm (b) was attributed to the methylene group [31]. The chemical shifts at 3.37 ppm was attributed to the protons of –O–CH_2_– group (c). The peak at 2.84 ppm was assigned to the protons of the hydroxyls which indicated that the epoxy group in the PGMA was opened up. Those chemical shifts at 6.02 (h), 6.35 (g), 6.57 (e), 6.89 (j), 7.12 (k) and 7.32 (i) ppm were assigned to the aminopyridine [32]. The molar ratio of the APD to PGMA–APD was 3:1 calculated by data from peak areas.

### Morphology and stability of both assemblies

**Figure 3:**
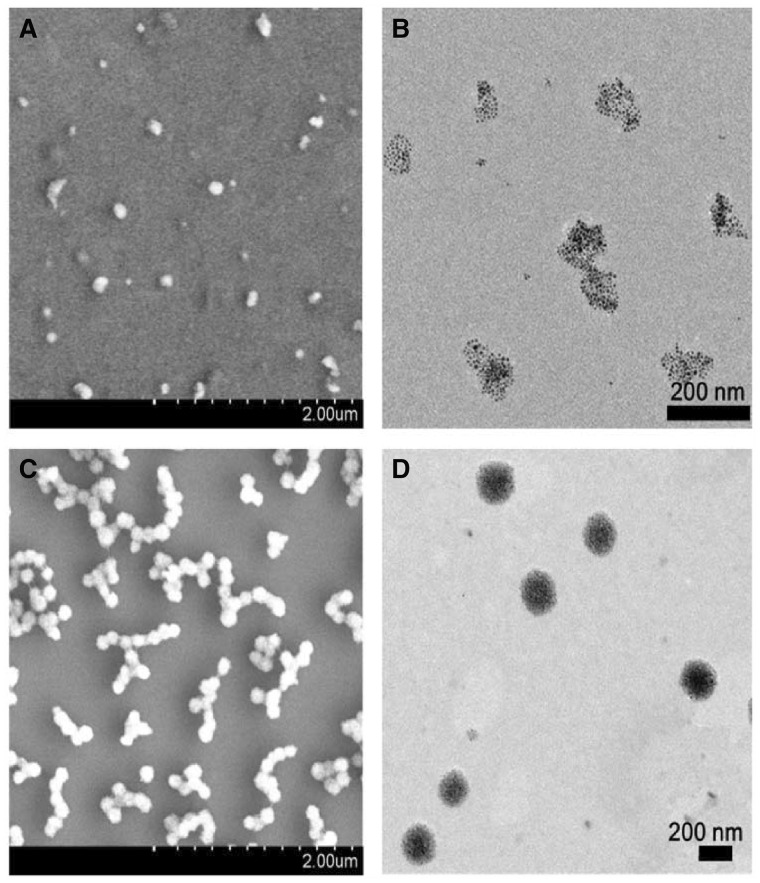
SEM and TEM images of the self-assemblies (A and B) and co-assemblies (C and D)

**Figure 4:**
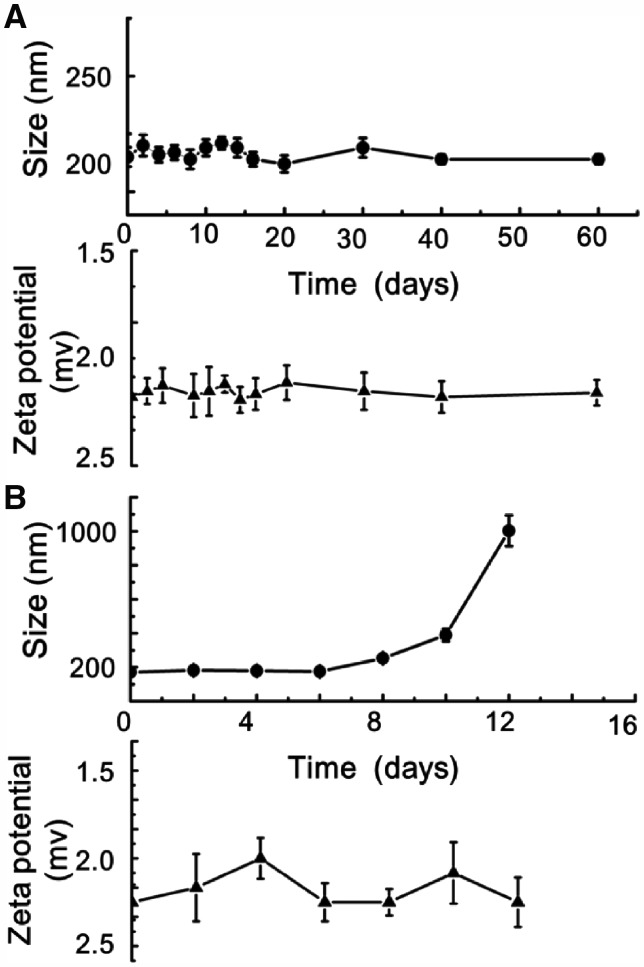
The corresponding size/zeta potential of self-assemblies (A) and co-assemblies (B) changed over time

As the functional components, Fe_3_O_4_@AJNPs were able to self-assemble into particles or co-assemble with the complementary substances. Therefore, the self-assembly behavior of Fe_3_O_4_@AJNPs and their co-assembly behavior with PGMA–APD were studied by TEM, SEM and DLS. Due to mutual attraction of the hydrophobic parts on the particles spontaneously [33], self-assembly of the Fe_3_O_4_@AJNPs generated particulate structures, and their average diameter detected by DLS (Supplementary Fig. S1), SEM (Fig. 3A) and TEM (Fig. 3B) was found to be 160 ± 13.7 nm. Specially, the Fe_3_O_4_@AJNPs showed random distribution in the self-assemblies. In contrast, co-assembly of Fe_3_O_4_ AJNPs and PGMA-APD resulted in the formation of monodisperse spherical superstructures (210 ± 22.5 nm in size) with an orderly arrangement of the Fe_3_O_4_@AJNPs (Fig. 3C and D). It was obvious that the co-assemblies had more orderly spherical superstructures than those with the self-assemblies. Stability of either the self-assemblies or co-assemblies was monitored by the variations of size and zeta potential of corresponding assembled particle over time (Fig. 4). Different from the hydrophobic interactions of the self-assemblies, host-guest interactions of the APD on the PGMA chains and β-CD on the Fe_3_O_4_@AJNPs were the main driving force to form the co-assemblies. Therefore, the binding strength of the Fe_3_O_4_@AJNPs in the co-assemblies was much stronger than that in the self-assemblies. As a result, stability of these co-assemblies was greatly improved, as one could see their uniform size distribution in deionized water even up to 2 months (Fig. 4A). However, the self-assemblies underwent an obvious size increase and precipitated within 2 weeks (Fig. 4B). This further demonstrated that the introduction of the PGMA-APD into Fe_3_O_4_@AJNPs can improve the stability of the co-assemblies.

### pH response and re-assembly behavior of co-assembly

**Figure 5:**
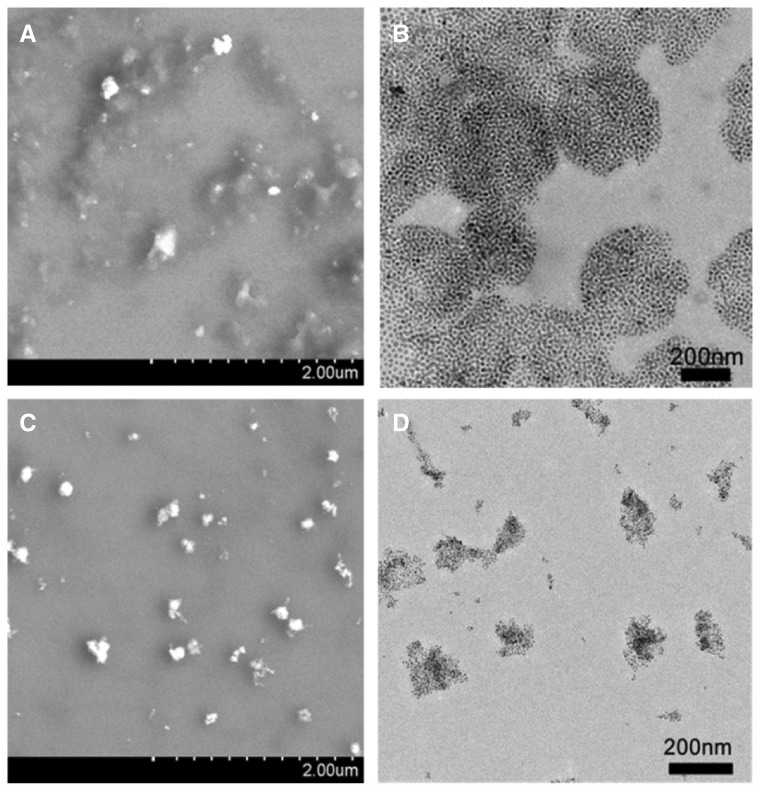
SEM and TEM images for the co-assemblies at pH 5.7 for 30 min (A and B) and 2 h (C and D)

**Figure 6:**
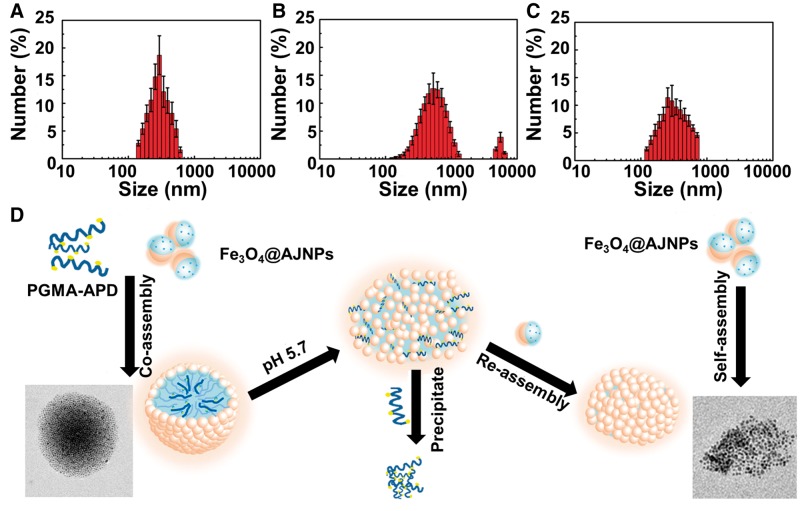
DLS size distribution data for the co-assemblies at pH 5.7 for 0 min (A), 30 min (B) and 2 h (C). (D) the schematic diagram of acid-triggered disassembly and reassembly process of co-assemblies

As we known that the assembly behavior of AJNPs in water was leaded by the mutual attraction of the hydrophobic parts on the particles spontaneously, which was irreversible [34]. Superstructures assembled by Fe_3_O_4_@AJNPs are static and have no environment stimuli-responsive behavior which was undesired in many fields like drug delivery and bio-separation. In this study, we firstly report the pH-responsive host-guest interactions between β-CD and APD to construct pH-responsive co-assemblies superstructures of Fe_3_O_4_@AJNPs. As one of the main building blocks, APD played an important role in controlling the stimuli-responsive properties of the co-assemblies due to the acid-liable disassembly process. In order to observe the direct disassembly process, the supernatant of co-assemblies solution when the environmental pH was adjusted to 5.7 for 30 min and 2 h, was investigated by SEM, TEM and DLS. As shown in Fig. 3, the steady particulate formations with an average diameter of 210 ± 22.5 nm (Fig. 6A) were constructed above pH 7. Swollen and/or ruptured assemblies with a broader size distribution were observed within 30 min when the pH was adjusted to 5.7 (Fig. 5A and B). In addition, a new peak at 5000 ± 362.5 nm appeared in the DLS results (Fig. 6B). Moreover, this change could be easy visualized, as shown in Supplementary Fig. S2A, where the suspension of the co-assembly system became muddy after adjusting pH value from 7.4 to 5.7. On the contrary, similar phenomenon was not found in the self-assembly system (Supplementary Fig. S2B). After the pH was adjusted to 5.7 for 2 h, the precipitate was collected and confirmed as PGMA–APD by FTIR (Supplementary Fig. S3). The characteristic absorption peaks representing pyridine ring (1617, 1516 and 1455 cm^−1^) of the precipitate were in accordance with that of PGMA–APD. Furthermore, the remaining particles in the supernatant were characterized by SEM, TEM and DLS. Interestingly, the results showed a similar morphology (Fig. 5C and D) and hydrodynamic diameter (Fig. 6C) to those self-assemblies of Fe_3_O_4_@AJNPs. The results suggested that during the acid-induced disassembly process (Fig. 6D), the nitrogen atoms in APD were protonated—in turn, this destroyed the inclusion complexes of β-CD and APD, leading to disassembly of the superstructures. As a result, the hydrophobic PGMA–APD chains lacking of interactions with the Fe_3_O_4_ @AJNPs and were released into water and precipitated. The remaining Fe_3_O_4_@AJNPs re-assembled into self-assemblies that were no longer pH-responsive.

**Figure 7:**
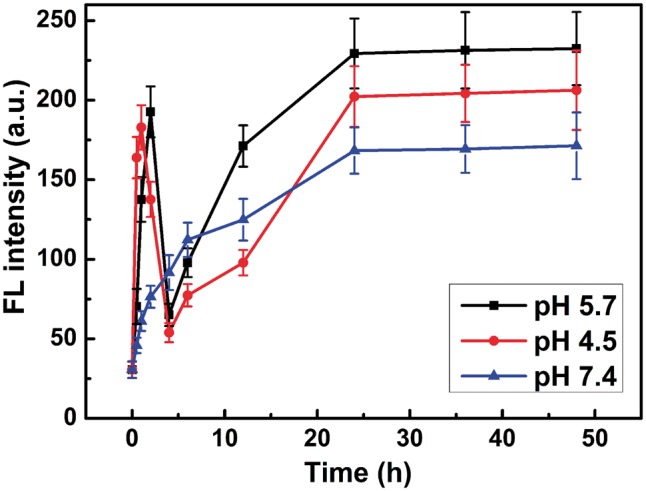
The fluorescence intensity of DOX-loaded co-assemblies at 558 nm changed with times in pH 5.7, 4.5 and 7.4 buffer solutions

To visualize this process more clearly, hydrophobic doxorubicin (DOX) molecules were encapsulated in co-assemblies and used as a fluorescent probe to monitor the disassembly process of the co-assemblies and re-assembly process of Fe_3_O_4_@AJNPs. As shown in Supplementary Fig. S4, the diameter of DOX-loaded co-assemblies was 235 ± 21 nm. The concentration of DOX-loaded co-assemblies in the aqueous solution was adjusted to 1.5 mg ml^−1^ and dispersed in buffers with pH 7.4, 5.7 and 4.5, respectively. The fluorescence spectra of DOX-loaded co-assemblies (Supplementary Fig. S5) and the curve of max emission peaks at 558 nm (Fig. 7) changed with time in different pH buffer solutions was acquired. Without pH stimulus, the fluorescence intensity of DOX-loaded co-assemblies slowly increased with time due to the DOX release caused by the dissolution and diffusion. While at pH 5.7 or 4.5, fluorescence intensity increased in the first, then decreased steeply to the relatively low value (73.4 ± 7.8 a.u. for pH 5.7; 58.3 ± 6.4 a.u. for pH 4.5), finally slowly increased to a maximum value (232.5 ± 22.5 a.u. for pH 5.7; 204.6 ± 19.1 a.u. for pH 4.5). These phenomenons confirmed the process of pH-stimulated disassembly of built nanospheres. More specifically, the initial weak fluorescence intensity was attributed to the DOX encapsulation in the co-assembly interiors [35, 36]. Then pH-stimulus caused the disassembly of DOX-loaded co-assemblies, leading to a burst release of DOX. After PGMA–APD chains precipitation, the remained Fe_3_O_4_@AJNPs reassembled to form self-assemblies, further resulting in re-encapsulation of DOX which caused the steeply decrease in fluorescence intensity. Since the self-assemblies of Fe_3_O_4_@AJNPs had no response to environmental pH conditions, the DOX loaded in re-assemblies released slowly for the above mentioned process as a consequence of cargo dissolution and diffusion.

### Saturation magnetization and the ability of re-assembly

**Figure 8:**
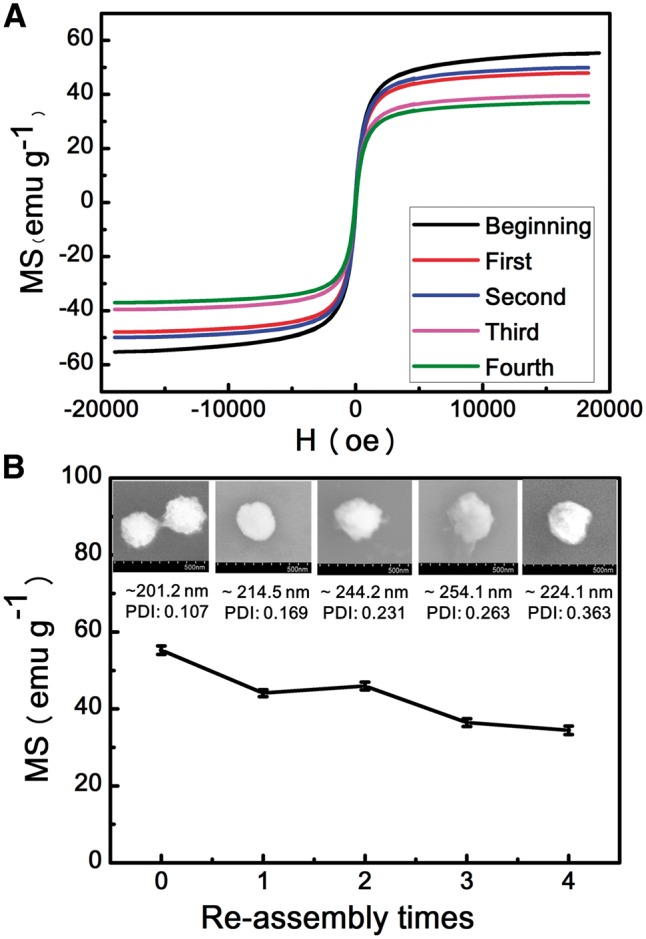
(A) The magnetization curves of co-assembles from different re-assembly times. (B) The SEM images, size and saturation ms value of co-assembles for different re-assembly times

It was suggested that Fe_3_O_4_ would be corroded by H^+^ [37]. Therefore, the superparamagnetism of Fe_3_O_4_@APJNs might be decreased. However, the pH variation was proven to be the main factor to stimulate these assembled superstructures in our work. On this basis, investigation of the magnetic properties of Fe_3_O_4_@APJNs and their reusability would be of great importance. Briefly, after PGMA-APD precipitation, the magnetically isolated Fe_3_O_4_@APJNs were re-used to create new co-assemblies with additional PGMA–APD polymers. Magnetic hysteresis loops, general morphologies and a saturation Ms value of the newly formed co-assemblies were examined. Corresponding magnetization curve was shown in Fig. 8A. When the field was zero, no remnant magnetic moment could be recorded, revealing the excellent superparamagnetic property of all these co-assemblies, even though Fe_3_O_4_@APJNs were re-used for several times. Related morphology, diameter and saturation Ms value were recorded (SEM, DLS and VSM) and shown in Fig. 8B. After four rounds of recycling, these re-used Fe_3_O_4_@APJNs could still build up steady nanoparticles with uniform size distribution together with the complementary polymer PGMA–APD. These results showed that the assembly capability of the Fe_3_O_4_@APJNs was not influenced by the environmental pH value. On the other hand, acid condition indeed affect magnetic property of the Fe_3_O_4_@APJNs, as one could find, saturation magnetization (Ms) value of the co-assemblies decreased slowly with recycle times. It should be noted, despite all this Ms decrease, recycled Fe_3_O_4_@APJNs still possessed higher Ms value than the previously reported ones. [38, 39] These co-assemblies also showed fast magnetic response and can be quick separated in 15 min with an ordinary commercial magnet.

### The cytocompatibility *in vitro*

**Figure 9:**
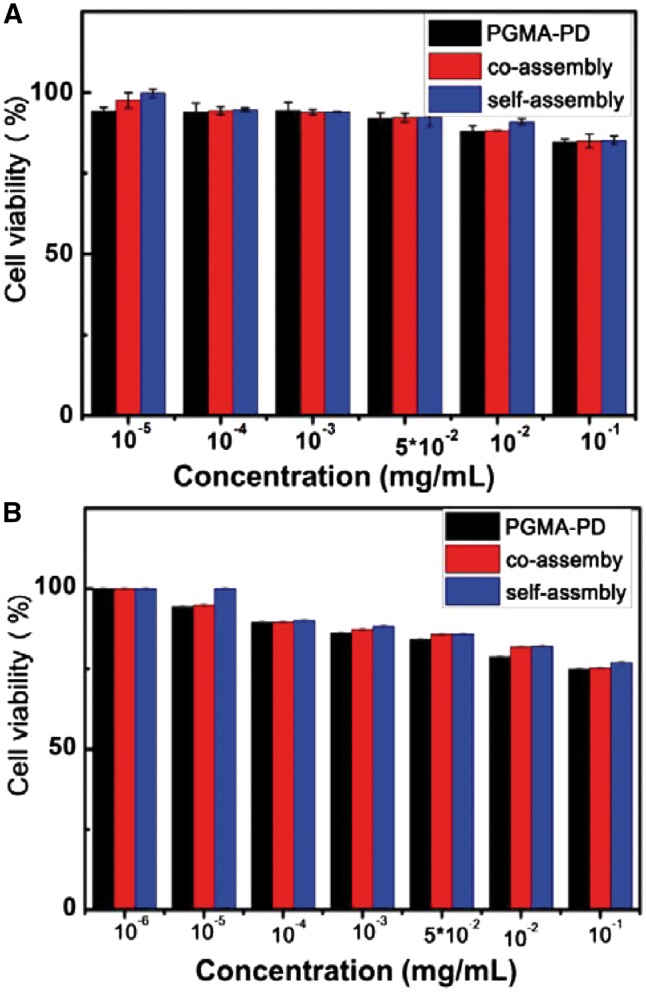
The cell viability of 3T3 cells (A) and HepG2 (B) cells incubated with co-assemblies, self-assemblies and PGMA–PD separately, examined by the CCK-8 assay

As a pH responsive system holding great potentials being used for drug delivery, these co-assemblies should have good biocompatibility. In this work, their cytocompatibility *in vitro* was studied preliminary. 3T3 and HepG2 cell lines were incubated with the co-assemblies, self-assemblies and PGMA-PD, respectively. The cell viability was assessed by the CCK-8 assay. As shown in Fig. 9, the cell viabilities of both 3T3 (Fig. 9A) and HepG2 cells (Fig. 9B) were over 80% when incubated with these materials, even at 100 μg/ml suggesting good cytocompatibility.

## Conclusions

In summary, we proposed for the first time, a strategy using Fe_3_O_4_@AJNPs together with a functional polymer to construct pH-stimuli responsive co-assemblies through host-guest interactions. SEM, TEM and DLS studies of self-assemblies built by Fe_3_O_4_@AJNPs and co-assemblies constructed with Fe_3_O_4_@AJNPs and PGMA–APD indicated that the co-assemblies had a more orderly and stable spherical superstructures than the self-assemblies. Moreover, the pH-responsive property of the co-assemblies was confirmed, and SEM, TEM, DLS monitored the disassembly process along with release of a fluorescent cargo. The Fe_3_O_4_@AJNPs had excellent reusability and could reassemble with additional PGMA-APD to build co-assemblies with a uniform morphology for several times. Third, these pH-sensitive co-assemblies showed excellent cytocompatibility *in vitro*. Overall, these pH-sensitive co-assemblies with a uniform morphology, high stability, good magnetic responsiveness and cytocompatibility have potential applications in intelligent nanomaterials and biomedical fields.

## Supplementary Material

Supplementary Data
