# Supplementary material for: pH-responsive superstructures prepared via the assembly of Fe3O4 amphipathic Janus nanoparticles
Source: Regen Biomater. 2018 Jul 12;5(5):251–9. doi: 10.1093/rb/rby016 (PMC6184715; doi:10.1093/rb/rby016)
Supplement: Supplementary Data [file rby016_supporting_information.docx]

**Supporting Information**

**pH-responsive superstructures prepared via the assembly of Fe_3_O_4_ amphipathic Janus nanoparticles**

Cai Shuang, Luo Bin, Zhan Xiaohui, Zhou Xiaoxi, Lan Fang, Yi Qiangying*, Wu Yao*

*National Engineering Research Center for Biomaterials, Sichuan University, Chengdu, Sichuan 610064, P.R. China*





Figure S1. The hydrodynamic diameter of the self-assemblies.


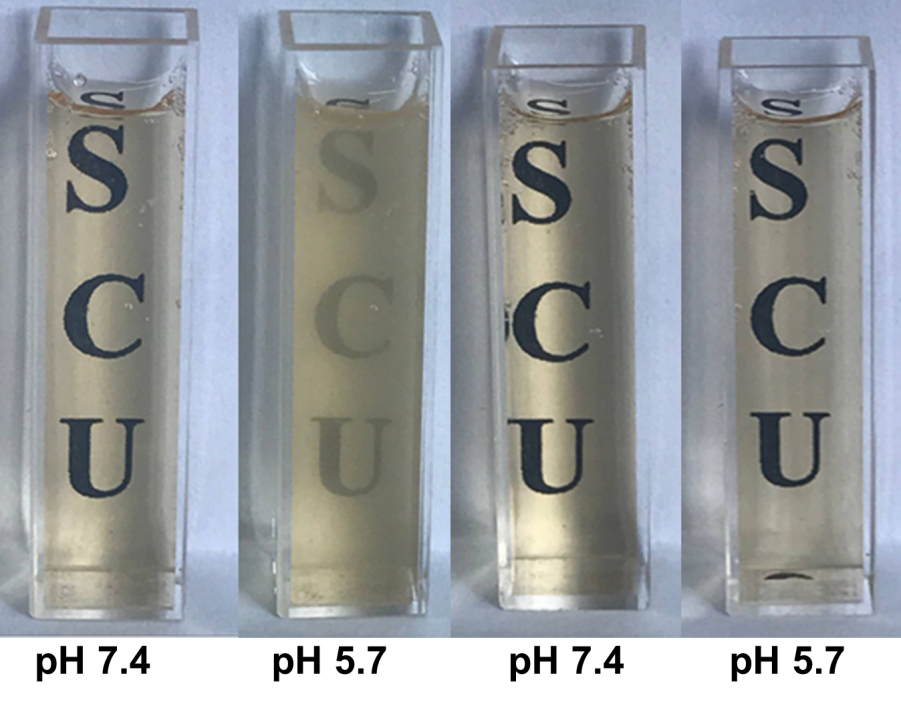


Figure S2 The [visualized](javascript:void(0);) images of co-assemblies (A) and self-assemblies (B) suspensions at pH 7.4 and pH 5.7.

**
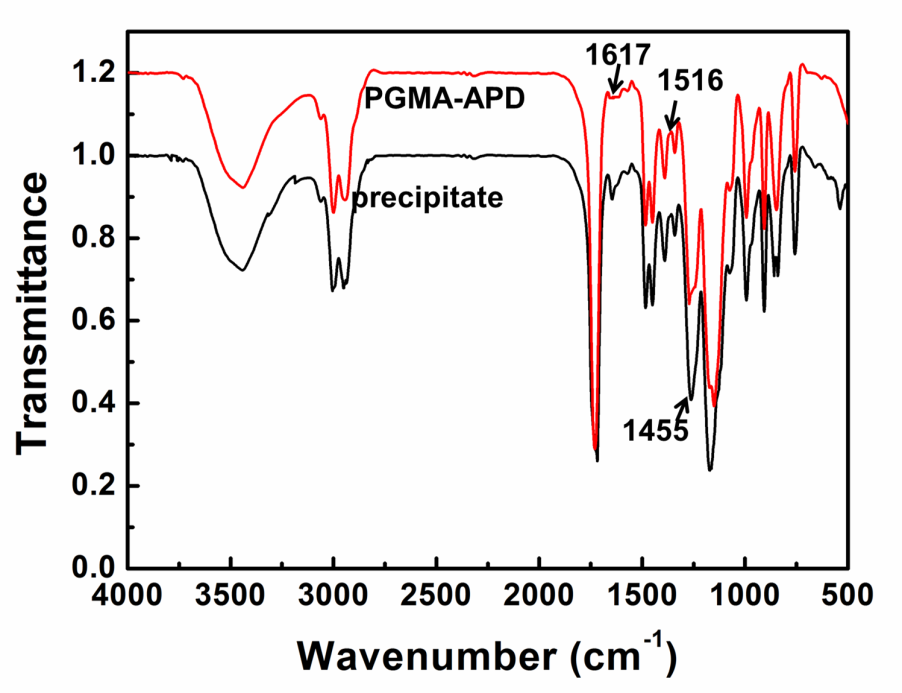
**

Figure S3 FTIR spectra of PGMA-PD and precipitate obtained from co-assemblies in acid circumstance.





Figure S4. The hydrodynamic diameter of the DOX-loaded co-assemblies.


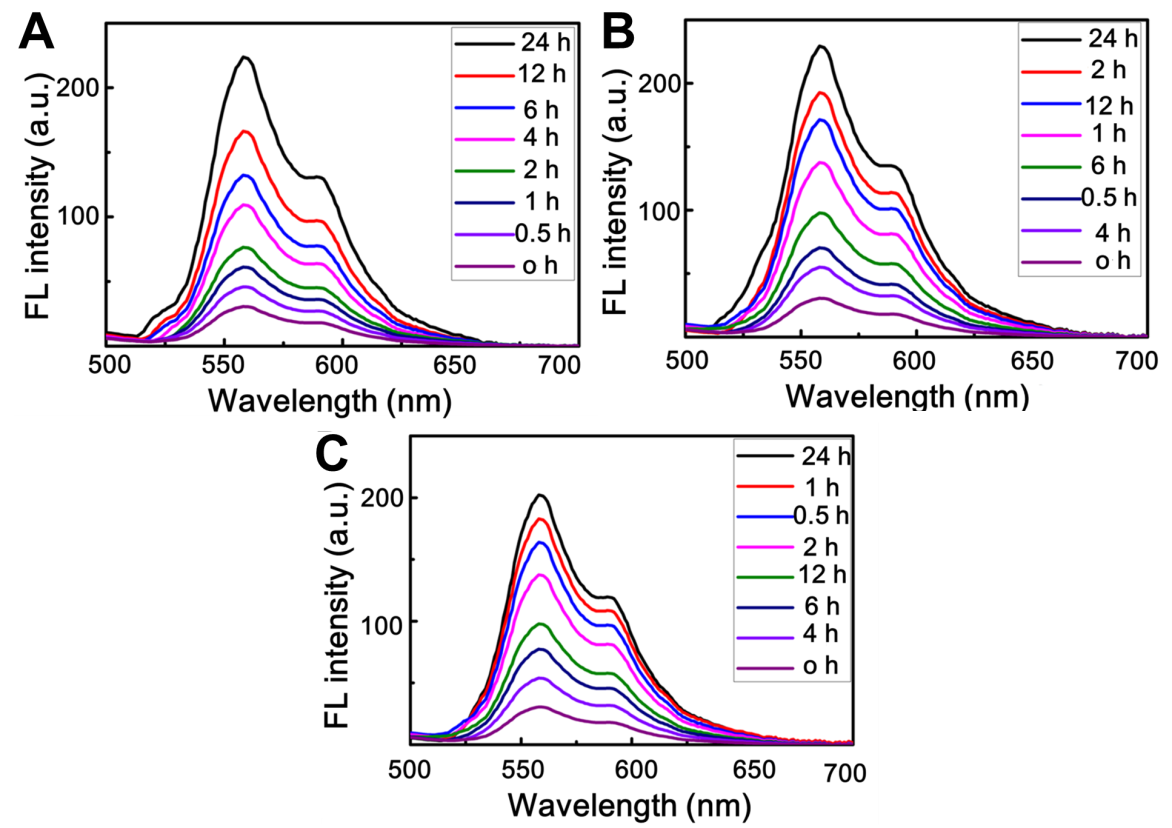


Figure S5. Fluorescence spectra of DOX-loaded co-assemblies in pH 7.4 (A), pH 5.7 (B) and pH 4.5 (C) buffer solutions at room temperature.
